# Robust CRISPR/Cas9 Genome Editing of the HUDEP-2 Erythroid Precursor Line Using Plasmids and Single-Stranded Oligonucleotide Donors

**DOI:** 10.3390/mps1030028

**Published:** 2018-07-30

**Authors:** Gemma Moir-Meyer, Pak Leng Cheong, Aude-Anais Olijnik, Jill Brown, Samantha Knight, Andrew King, Ryo Kurita, Yukio Nakamura, Richard J. Gibbons, Douglas R. Higgs, Veronica J. Buckle, Christian Babbs

**Affiliations:** 1 MRC Molecular Haematology Unit, MRC Weatherall Institute of Molecular Medicine, University of Oxford, Oxford OX3 9DS, UK; gemma.moir.meyer@gmail.com (G.M.-M.); cheong80@gmail.com (P.L.C.); aude-anais.olijnik@ndcls.ox.ac.uk (A.-A.O.); jill.brown@imm.ox.ac.uk (J.B.); andrewjohnking@gmail.com (A.K.); richard.gibbons@imm.ox.ac.uk (R.J.G.); doug.higgs@imm.ox.ac.uk (D.R.H.); veronica.buckle@imm.ox.ac.uk (V.J.B.); 2 Wellcome Trust Centre for Human Genetics, Oxford University, Oxford OX3 7BN, UK; sknight@well.ox.ac.uk; 3 Department of Research and Development, Central Blood Institute, Japanese Red Cross Society, 1-1-3 Shibadaimon, Minato-ku, Tokyo 105-8521, Japan; r-kurita@jrc.or.jp; 4 RIKEN BioResource Research Center, Koyadai 3-1-1, Tsukuba 305-0074, Japan; yukio.nakamura@riken.jp

**Keywords:** CRISPR/Cas9, HUDEP-2 cells, homology directed repair, anaemia

## Abstract

The study of cellular processes and gene regulation in terminal erythroid development has been greatly facilitated by the generation of an immortalised erythroid cell line derived from Human Umbilical Derived Erythroid Precursors, termed HUDEP-2 cells. The ability to efficiently genome edit HUDEP-2 cells and make clonal lines hugely expands their utility as the insertion of clinically relevant mutations allows study of potentially every genetic disease affecting red blood cell development. Additionally, insertion of sequences encoding short protein tags such as Strep, FLAG and Myc permits study of protein behaviour in the normal and disease state. This approach is useful to augment the analysis of patient cells as large cell numbers are obtainable with the additional benefit that the need for specific antibodies may be circumvented. This approach is likely to lead to insights into disease mechanisms and provide reagents to allow drug discovery. HUDEP-2 cells provide a favourable alternative to the existing immortalised erythroleukemia lines as their karyotype is much less abnormal. These cells also provide sufficient material for a broad range of analyses as it is possible to generate in vitro-differentiated erythroblasts in numbers 4–7 fold higher than starting cell numbers within 9–12 days of culture. Here we describe an efficient, robust and reproducible plasmid-based methodology to introduce short (<20 bp) DNA sequences into the genome of HUDEP-2 cells using the clustered regularly interspaced short palindromic repeats (CRISPR)/CRISPR associated protein 9 Cas9 system combined with single-stranded oligodeoxynucleotide (ssODN) donors. This protocol produces genetically modified lines in ~30 days and could also be used to generate knock-out and knock-in mutations.

## 1. Introduction

Studying disease pathogenesis affecting terminal erythroid differentiation requires model cellular systems capable of generating sufficient material to allow genomic, transcriptomic, proteomic and cell biology approaches. Analysis of erythroblasts from circulating progenitors derived from the peripheral blood of healthy individuals and patients can provide invaluable insights into normal and diseased erythropoiesis and is often considered to be the gold standard. However, limited patient availability, a lack of specific antibodies and polygenic background effects that obscure core phenotypic abnormalities are all limitations of this approach and analysis of genetically modified cell lines may help to circumvent these issues. Additionally, the use of patient cells often provides insufficient material for many approaches.

To deal with these problems, a number of cell lines have been used to study terminal erythroid differentiation in the laboratory. These include the human erythroleukaemia cell lines termed K562 and HEL [1,2] and the mouse equivalent, termed MEL [3]. Study of these cell lines in conjunction with small interfering RNA (siRNA) mediated knockdown of specific genes has provided insight into normal and aberrant terminal erythroid differentiation, however, these lines are extremely aneuploid and harbour chromosome rearrangements that hinder genomic manipulation and may adversely affect terminal differentiation [4]. Recently it has become possible to generate and genetically manipulate induced pluripotent stem cells (iPSCs) from healthy individuals and patients, however, maintenance of these cells is time-consuming and costly and when transdifferentiated into erythroblasts iPSCs express mainly embryonic and fetal globins [5].

Study of erythroid differentiation using primary cells obtained from mice continues to be extremely informative, however, there is considerable effort and cost associated with generating and breeding mice lines harbouring specific mutations and expressing tagged proteins and it is imperative to replace animal models with alternative systems where possible [6]. Additionally, although animal models of human disease can be very informative it is not uncommon to find that human diseases are not accurately recreated in mouse models. One example of this is the *Sec23b* mutant mouse which does not recapitulate a type of anaemia termed congenital dyserythropoietic anaemia type II CDA-II, known to be caused by biallelic mutation of *SEC23B* in humans [7].

The recently published immortalised line derived from Human Umbilical Derived Erythroid Precursors and termed HUDEP-2 cells [8] offers an alternative system in which to study erythroid differentiation. HUDEP-2 cells express predominantly adult globins and are capable of robust in vitro erythroid differentiation and therefore are an excellent resource for modelling human terminal erythropoiesis. Analysis of wild-type and genetically modified HUDEP-2 cells offers insight into a range of erythroid disorders including the haemoglobinopathies and congenital forms of anaemia. In the case of α- and β-thalassemia and sickle-cell anaemia, genome editing strategies and their effects may be tested in HUDEP-2 cells and valuable information about the genomic changes associated with these diseases, such as chromatin organisation and effects on gene expression may be gained (for a striking example see Wienert et al. [9]). In the case of the congenital anaemias, edited cells may be used to offer insight into the pathogenesis of these disorders and as a reagent to screen for potential novel therapies. In this work we use the rare anaemia termed congenital dyserythropoietic anaemia type I CDA-I, which is caused by loss of function mutations in either of the genes *CDAN1* or *C15ORF41*, [10,11] as an example with which to demonstrate the efficacy and utility of genome editing. To date, study of the pathogenesis of this disease has been hindered by variable phenotypic abnormalities, difficulty in obtaining patient samples and a lack of specific antibodies.

**Figure 1:**
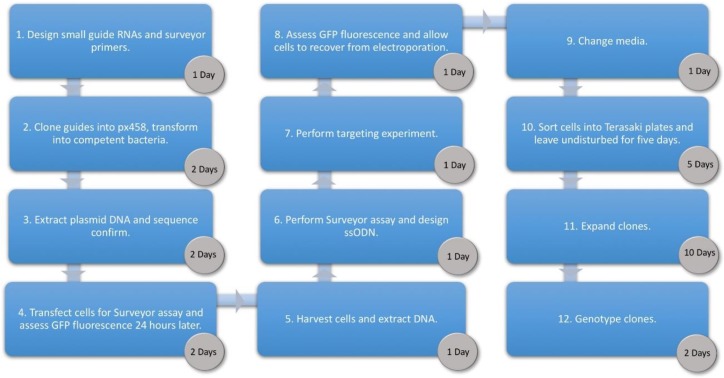
Workflow showing each phase of the Human Umbilical Derived Erythroid Precursor 2 (HUDEP-2) targeting protocol and the time required to complete each step. px458, plasmid #48138 from Addgene, Cambridge, Massachusetts, U.S.A.; GFP, green fluorescent protein; ssODN, single-stranded oligodeoxynucleotide.

There are some advantages to using ribonucleoprotein (RNP) delivery of Cas9 for inducing DNA breaks in terms of cutting efficiency and reduced off target effects as the Cas9 protein is cleared from the cell more quickly than when a plasmid-based expression system is employed [12]. If the user prefers to use RNP based editing, we would recommend using the protocol described in [13] combined with the sorting and clonal expansion protocol described here. It should be noted, however, there may be inter-batch and inter-supplier variability in Cas9 protein efficacy. Additionally, a current limitation of the RNP approach is a lack of flexibility to utilise the increasing array of Cas9 based approaches such as editing of the epigenome and base editing (for examples see Wang et al. [14]). Therefore, here we report a robust and efficient protocol delivering Cas9 via plasmids and the template for homology directed repair (HDR) of cleaved DNA using single-stranded oligodeoxynucleotide (ssODN) donors for generating cellular model systems, see Figure 1 for an overview. This protocol is flexible as it allows novel Cas9 variants of choice to be used, as long as the relevant Cas9 expressing plasmid can be generated or obtained. The protocol reported here allows generation of stable clones of genetically modified HUDEP-2 cells in ~30 days, analysis of which will greatly accelerate research into cellular processes, gene regulation and drug discovery in normal and abnormal erythroid differentiation.

## 2. Experimental Design

The desired targeting site should be identified and small guide RNAs (sgRNAs) designed. Care should be taken to select a region in which the copy number is normal, otherwise this should be accounted for during the analysis and repeated rounds of targeting may be required. If homology directed repair with a donor template is required, the Cas9 cut site should be within 20 bp of the required integration site. One limitation of the approach detailed here is that the *Streptococcus pyogenes* Cas9 protein used in this protocol requires an NGG or an NAG motif at the 3′ of its recognition sequence in order to cut. If there is no NGG or NAG within 20 bp of the required targeting site, then Cas9 proteins from alternative bacterial species (such as *Neisseria meningitidis* or *Staphylococcus aureus*) should be considered [14]. This protocol uses a single plasmid that contains sequence encoding the RNAIII polymerase U6 promoter driving expression of the sgRNA and a chicken β hybrid (Cbh) promoter driving expression of Cas9 and enhanced green fluorescent protein EGFP proteins separated by a T2A sequence.

The efficiency of sgRNA cutting should be tested in human embryonic kidney (HEK) 293T cells or any other easily transfectable cell line available to the user and the plasmid inducing the most efficient cutting of the target locus selected (see Section 4 Expected Results for further details). HUDEP-2 cells may also be used for this step if the user wishes to establish the cell-line specific cutting efficiency of each sgRNA. Once the exact cut site has been validated a ssODN should be designed with the homology arms centered on the cut site. One limitation of using ssODNs is that relatively short sequences may be introduced. This may be overcome by generating a donor plasmid, or an adeno-associated virus (AAV) vector, which may include a selectable marker to improve efficiency (see Bak et al. [13]). The validated Cas9-T2A-EGFP plasmid and ssODN should be nucleofected into the HUDEP-2 cells and after 48 h EGFP positive cells are single-cell sorted into microplates. Clones should be expanded and screened and positive clones taken forward for analyses. It should be noted that HUDEP-2 cells express variable levels of Kusabira Orange fluorescent protein and as such Cas9 plasmids using mCherry or other red-fluorescent protein should be avoided. If the gene targeted is essential for HUDEP-2 cell survival there may be no positive clones recovered and the experimental design should be reconsidered. 

### 2.1. Targeting Design and Human Umbilical Derived Erythroid Precursor 2 Cytogenetics

An important step in considering the design of the experiment is to check the copy number of the intended target region. As there may be a degree of clonal variation in the karyotype of this cell line it is advisable to ascertain the chromosome number prior to starting experiments on a particular line.

Chromosome counts to characterise the karyotype of unmodified HUDEP-2 cells reveal a modal chromosome number of 51, XY (6/10 metaphases) with a range of 49–53 chromosomes. Supernumerary chromosomes comprised a larger marker chromosome and four smaller chromosomes. Array comparative genomic hybridization (aCGH) (using an Illumina InfiniumOmniExpress-24v1-2_A1 Beadchip (see Appendix A for details) reveal trisomies of chromosomes 6, 17 (partial ~30–80 Mb), 18 (partial ~18–78 Mb), 19 and 21 (partial ~14–48 Mb). There is also variable gain of material along the length of chromosome 8 suggesting that the large marker may consist of rearranged chromosome 8 material. There are also smaller regions of loss of heterozygosity (Table 1).

Once the locations of each desired modification have been identified suitable sequences for sgRNAs should be identified. In this protocol we recommend use of short guides of 18 nucleotides to minimise the homology available for off-target binding (see Discussion). To design each guide, select 18 base pairs immediately 5′ of the closest located protospacer adjacent motif (PAM) site, this is NGG for *S. pyogenes* Cas9 although NAG may also be used with reduced efficiency (see reference [14]). In our experience integration of a donor is most successful when Cas9 cut sites are within 20 bp of the desired integration site, note that *S. pyogenes* Cas9 cuts between the 3rd and 4th bases 5′ of the PAM. To allow redundancy and subsequent selection of the most efficient site select two PAMs closest to the desired cut site and test cutting efficiency at both sites by Surveyor assay. We recommend sgRNA sequences be selected to minimise the possibility of off-target hybridisation by checking for other regions of homology using the basic local alignment tool BLAT [15] and where alternatives are available the sequence with the least homology to off-target sites should be selected.

### 2.2. Materials

#### 2.2.1. sgRNA Preparation

- pX458 & LKO1.5 (Addgene, Cambridge, MA, USA) [16]
- T4 Ligase 400U/μL & buffer (NEB, Ipswich, MA, USA; Cat. no.: M0202S).
- Oligonucleotides encoding sgRNA (Integrated DNA Technologies (IDT), Leuven, Belgium).
- *Bbs*I FastDigest enzyme & buffer (ThermoFisher Scientific, Hemel Hempstead, UK; Cat. no.: FD1014).
- Midi Plasmid *Plus* Midi kit (Qiagen, Hilden, Germany; Cat. no.: 12943).
- Nuclease free water (Ambion, Foster City, CA, USA; Cat. no. AM9937).
- DH10B competent *Escherichia coli* bacteria (ThermoFisher Scientific; Cat. no.: 18297010).
- Surveyor Mutation Detection Kit (Integrated DNA Technologies; Cat. no. S100).
- jetPRIME transfection reagent (Polyplus-transfection, New York, NY, USA; Cat. no. 114-07).
- Proteinase K (Ambion; Cat. no.:2546).
- FastStart Taq DNA Polymerase deoxynucleotide triphosphates (dNTP) pack (Roche, Mannheim, Germany; Cat. no. 04738381001).

#### 2.2.2. Growth Medium

- Stemspan SFEM (Stem Cell Technologies, Vancouver, BC, Canada; Cat. No. 09650).
- Glutamax 2 mM (ThermoFisher Scientific; Cat. no.: 25030-024).
- Penicillin-Streptomycin (100 units Penicillin and 100 μg Streptomycin/mL) (ThermoFisher; Cat. no.: 15140-122).
- Human Stem Cell Factor (SCF) (50 ng/mL) (Peprotech, Rocky Hill, NJ, USA; Cat. no: 300-07).
- Erythropoetin (3 IU/mL) (Janssen-Cilag, High Wycombe, UK, 10,000 IU/mL pre-filled syringe).
- Dexamethasone (DEX) (330 μg/L [840 nM]) (Hameln, Gloucester, UK; Cat. no.: DEXA3.3).
- Doxycycline (DOX) (1 μg/mL) (Sigma Aldrich, St Louis, MO, USA; Cat. no.: D3447).
  Note: Doxycycline has limited stability at 37 °C and requires supplementing every 24 h. Alternatively, 2 μg/mL doxycycline can be used in place of 1 μg/mL, enabling media to be changed every 48 h.
- HUDEP-2 cells were provided as an ongoing collaboration and cultured as described in Reference [8].

#### 2.2.3. Freezing Medium

- 90% 0.4 μm filtered fetal bovine serum (Sigma Aldrich; Cat. no.: F7524).
- 10% dimethyl sulfoxide (DMSO) (Sigma Aldrich; Cat. no.: D2650).

#### 2.2.4. Transfection

- 2B Amaxa Human CD34 Cell Nucleofector Kit (Lonza, Köln, Germany; Cat. no.: VPA-1003).
- RAD51-stimulatory compound-1 (RS-1) (Sigma Aldrich; Cat. no.: R9782).
- Complete media as described in Reference [8].
- Doxycycline (Sigma Aldrich; Cat. no.: D3447).
- Hoechst 33258 (Invitrogen, Carlsbad, CA, USA; Cat. no.: H3569).

### 2.3. Equipment

- AMAXA Nucleofector 2B (Amaxa, London, UK).
- Terasaki plates (Greiner, Kremsmünster, Austria; Cat no.: 653102).
- Fluorescence Activated Cell Sorter.
- Programmable Thermocycler.

## 3. Procedure

### 3.1. Cloning Oligonucleotides for Small Guide RNAs Time for Completion: 3 Days, Approx. 1 h per Day

**Figure 2:**
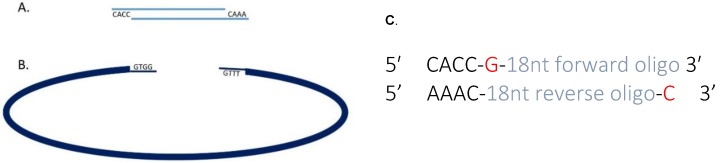
(**A**) Heteroduplexed small guide RNA with overhangs for ligation with *Bbs*I digested pX458; (**B**) *Bbs*I digested pX458 creates overhangs that are homologous to those on heteroduplexed sgRNAs; (**C**) oligo duplex design for a guide targeting the sense strand, the G/C shown in red is optional and may be added where the 5′ end of the sequence does not terminate in a guanine.

Oligonucleotides for use as sgRNAs should be obtained as 25 nmol scale desalted single-stranded oligonucleotides. Design sgRNA oligonucleotides to include 4-nucleotide 5’ overhangs (forward oligo 5′-CACC-3′; reverse oligo 5′-AAAC-3′) compatible with the *Bbs*I restriction enzyme site used to clone them into the pX458 plasmid [16] (Figure 2). Additionally, because the human U6 RNA polymerase III promoter in the pX458 plasmid preferentially transcribes sequences beginning with a guanine [17], sgRNA sequences that do not naturally include a 5′, “G” should include this nucleotide (Figure 2).

Clone oligonucleotides encoding sgRNAs into pX458 by heteroduplexing followed by the one-step protocol described by Cost [18]. Briefly, heteroduplex oligonucleotides by combining the reagents listed below in a single well of a microtiter plate and heating at 95 °C for 5 min before cooling to 12 °C at a rate of 0.1 °C per second using a thermocycler.

- 1 μL of 100 μM sgRNA oligonucleotide F.
- 1 μL of 100 μM sgRNA oligonucleotide R.
- 8 μL nuclease free water.

Perform enzymatic ligation assisted by nucleases (ELAN) reactions by combining the following reagents in a single well of a microtiter plate:

- 1 μL *Bbs*I 10× FastDigest buffer.
- 7.5 μL dH_2_O.
- 1 μL (0.5 μg) circular pX458 plasmid.
- 0.5 μL *Bbs*I Fast Digest enzyme.
- 1.25 μL 10× T4 DNA Ligase Buffer.
- 0.5 μL heteroduplexed oligonucleotides.
- 0.75 μL Ligase.
- Total volume = 12.5 μL.

Incubate ELAN reactions at 37 °C for 1 h before transforming into DH10B competent *E. coli* bacteria according to the manufacturer’s instructions. Clonally select and screen using the LKO1.5 forward primer in combination each specific sgRNA reverse oligonucleotide. Amplification conditions: 0.5 μM each oligonucleotide, 1 μL of bacterial culture (template), 200 μM dNTPs, 1× polymerase specific amplification buffer, one unit thermostable DNA polymerase combined in a total of 20 μL. Amplification reactions should be incubated at 95 °C for 3 min and then cycled 35 times at 95 °C for 30 s, 58 °C for 30 s and 72 °C for 30 s before a final incubation at 72 °C for 10 min. Correctly recombined clones should produce a discrete amplification product of ~100 bp, visible by agarose gel electrophoresis. Grow correctly recombined clones overnight in 50 mL LB broth supplemented with 0.1 mg/mL ampicillin and isolate plasmid DNA using the Plasmid *Plus* Midi kit (Qiagen) in accordance with the manufacturer’s directions.

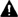
**CRITICAL STEP** It is important to elute plasmids in a small volume (50 μL) to achieve concentrations of 4–6 μg/μL.

### 3.2. Surveyor Assay. Time for Completion: 1 Day

**Figure 3:**
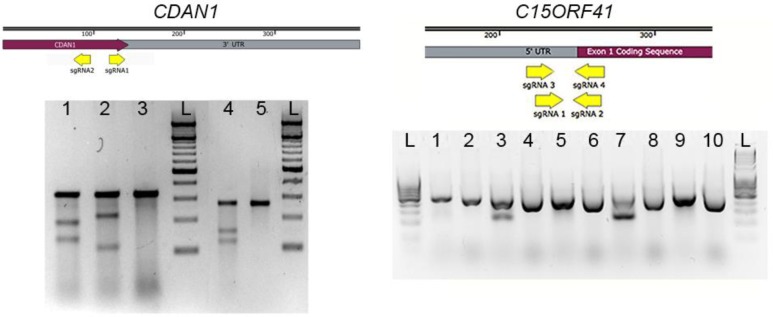
Expected outcome of surveyor assays. Upper, schematic of the 3′ region of *CDAN1* and 5′ region of *C15ORF41* showing the position of the sgRNAs (upper panels). Results of surveyor assays are shown (lower panels). *CDAN1* surveyor assay: 1. sgRNA 1; 2. sgRNA 2; 3. Control (amplification product not treated with nuclease); 4. Nuclease-treated positive control; 5. Nuclease-treated IDT negative control. *C15ORF41* surveyor assay: 1. sgRNA 1; 2. sgRNA 1 uncut; 3. sgRNA 2; 4. sgRNA 2 uncut; 5. sgRNA 3; 6. sgRNA3 uncut; 7. sgRNA 4; 8. sgRNA4 uncut; 9,10. -ve control (no sgRNA cut and uncut). Cutting efficiency is reflected by the intensity of lower bands relative to the full-length undigested amplification product, Image Lab (Bio-Rad, Hercules, CA, USA) was used to quantify band intensities.

To assess the efficiency with which each Cas9 plasmid cuts its target site, transfect a 35 mm well of HEK 293T cells (or other available cell line) with 2 μg of plasmid using jetPRIME reagent (or other transfection reagent of choice) in accordance with manufacturer’s instructions. Grow cells for 48 h post transfection and prepare genomic DNA (lyse cells for 2–4 h at 37 °C in a buffer containing 50 mM Tris pH 8.0, 1 mM ethylenediaminetetraacetic acid (EDTA), 0.5% Tween 20 and 60 μg/mL proteinase K. Heat inactivate proteinase K at 95 °C for 10 mins). Design oligonucleotides to generate amplification products of 500–800 bp around the target cut site and treat amplified DNA with Surveyor nuclease in accordance with the manufacturer’s directions. Analyse digestion products by agarose gel electrophoresis and use Cas9 plasmids that generate samples with the highest ratio of digested to undigested product to target HUDEP-2 cells. Example Surveyor assays are shown in Section 4 Expected Results (Figure 3).

### 3.3. Design of Oligonucleotide Donor. Time for Completion: 2 h

**Figure 4:**
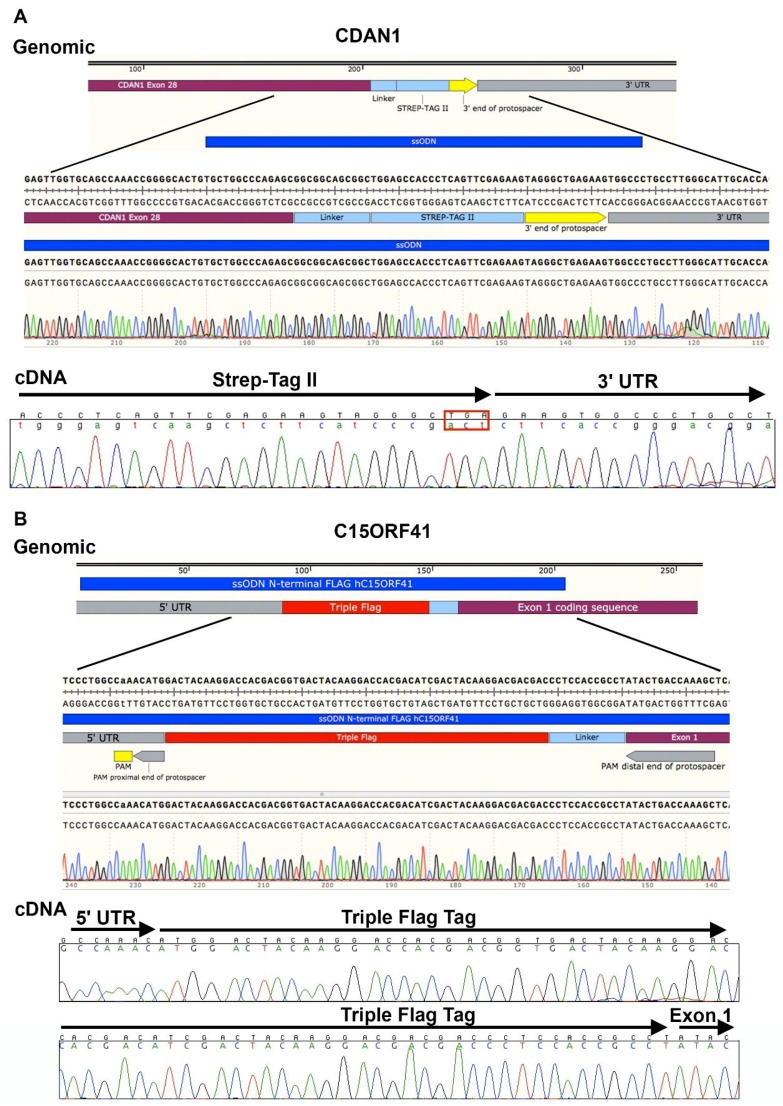
Expected results of targeting two endogenous loci. Schematic diagram (upper), annotation (middle) and chromatograms (lower) showing homozygous insertion of affinity purification tags into endogenous loci. (**A**) Insertion of a Strep-tag II at the 3′ end of *CDAN1* with chromatogram data to show correct integration into the genomic locus and of a correctly spliced complementary DNA (cDNA) product (the TGA stop codon is highlighted with a red rectangle). (**B**) Insertion of a triple FLAG-tag at the 5′ end of *C15ORF41* including chromatograms to show correct insertion at the endogenous locus and cDNA containing the tag. ssODN, single-stranded oligo deoxynucleotide.

To introduce short DNA sequences into endogenous loci, ssODNs may be used. Donors may be obtained from IDT (Leuven, Belgium) as Ultramers^®^ 199 bp in length. The donors used in our example (see Section 4 Expected Outcomes) included a 24 bp sequence encoding a Strep-tag [19] or FLAG-tag [20] and a four amino acid flexible linker: Gly-Gly-Ser-Gly for integration into the *CDAN1* or *C15ORF41* locus respectively. To prevent repeated cutting at the same locus, ssODNs included a silent mutation, which alters either the first or second “G” of the “NGG” PAM site required for Cas9 binding. Changing the PAM to NAG should be avoided where possible as *S. pyogenes* Cas9 may still be able to cut albeit with reduced efficiency. We found that homology arms equidistant from the cut site efficiently facilitated homology driven repair of the locus following the double strand break generated by the Cas9 protein. Additionally, we recommend the small molecule agonist of RAD51, RS-1, be used to promote repair of the double stranded break with homology directed repair [21]. Expected outcomes may be seen in Figure 4.

If required, heterozygous genotypes can be reliably generated with two rounds of editing. The first targeting experiment uses an ssODN to introduce a silent mutation in the PAM site. Due to the incomplete efficiency of biallelic targeting, numerous clones will have successful integration of the ssODN on one allele and an indel resulting from non-homologous end joining on the other allele. In the second experiment, the allele containing the indel is targeted specifically with a newly designed sgRNA. A ssODN which repairs the indel, disrupts the remaining PAM and introduces the desired mutation is transfected with the new sgRNA. 

### 3.4. Transfection of Human Umbilical Derived Erythroid Precursor 2 Cells. Time for Completion: 3 Days

Cells should be expanded for 5–7 days and be in an exponential growth phase prior to transfection. Change media to adjust density to 0.5 × 10^6^/mL 24 h prior to transfection and maintain wild-type untargeted cells in expansion phase for use as a negative control during the selection of GFP-positive clones.

#### 3.4.1. Day 1. Time for Completion: 3 h

1. Count cells.
2. Aliquot 7.5 μg of plasmid, 9 μL 200 μM ssODN, 18.8 μL CD34 kit supplement and 81.2 μL CD34 kit buffer into a microcentrifuge tube.
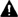
**CRITICAL STEP** The ratio of DNA to transfection reagents must be maintained as 1:10.
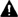
**CRITICAL STEP** Do not exceed 110 μL total volume in the cuvette.
3. Prepare the appropriate volume of media to resuspend cells at a density of 1 × 10^6^ per mL with 2 μg/mL DOX.
4. Aliquot 3.25 × 10^6^ cells into a tube and centrifuge at 270× *g* for 5 min.
5. Aspirate supernatant, resuspend the cells by flicking the tube then add >10mL of phosphate buffered saline (PBS) and centrifuge at 270× *g* for 5 min.
6. Aspirate PBS removing as much liquid as possible. This is important in ensuring an efficient transfection.
7. Resuspend cells pellets in buffer/supplement/plasmid/donor solution and transfer (<110 μL) into a Nucleofector Amaxa 2B cuvette by gently dispensing the cells down the side of the vessel between the metallic plates and without introducing any bubbles.
8. Nucleofect the cells by placing the cuvette in the Amaxa 2B Nucleofector using protocol U-08.
9. Immediately following nucleofection return the cuvette to tissue culture hood and add growth media (see Section 2.2 for description) containing RS-1 (if required) to the cuvette. Aim to dilute the buffer a minimum of 5:1 media:buffer but ideally 10:1 within the first minute post nucleofection. Keep 1 mL of resuspension volume to wash cuvette with after transferring the cells into a culture vessel.
10. Using the Pasteur pipette provided in the nucleofection kit, remove cells from the cuvette and gently triturate the solution to evenly distribute them.
11. Rinse the cuvette with the remaining 1 mL of media and add this to the culture vessel.
12. Resuspend the cells to a density of 1 × 10^6^ cells/mL in the prewarmed media with 2 μg/mL DOX
  **OPTIONAL STEP** If you are attempting to integrate a donor at the cut site it is possible to use the small molecule RS-1 to promote homology driven repair (0.75 μM final concentration in the cell resuspension media) [21,22]. RS-1 is a small molecule activator of RAD51, which is thought to be involved in finding a homologous repair template and facilitating strand exchange [23].

#### 3.4.2. Day 2. Time for Completion: 20 min

13. Assess cells for fluorescence using a microscope. Expect 30–50% GFP-positive cells.

#### 3.4.3. Day 3. Time for Completion: 30 min

14. Count cells and perform a full media change by centrifuging cells at 270× *g* for five minutes and resuspending cells in growth medium at a density of 1 × 10^6^ cells/mL. RS-1 is no longer required.

### 3.5. Cell Sorting. Time for Completion: 6 h 

#### Day 4

15. Using fluorescence activated cell sorting (FACS) sort single cells into each well of a 60-well microtiter plate (Terasaki plate). Each well should be prefilled with 20 μL media containing 2 μg/mL DOX and maintained in an incubator until the cells have been prepared for sorting.
16. Prepare both the transfected cells and 1 × 10^5^ untransfected cells by centrifugation at 270× *g* for five minutes and washing once with PBS. After recentrifugation and discarding PBS, resuspend the cell pellet in complete media with 2 μg/mL DOX and 1 μg/mL Hoechst 33258 to a density of 1 × 10^6^–5 × 10^6^ per mL as appropriate depending on the FACS machine and preferred flow rate. Hoechst is used to differentiate live/dead cells during sorting.
17. Set gate for GFP-positive cells based on the untransfected population and sort single GFP-positive cells into each well of 10 Terasaki plates.
18. Once cells have been sorted, transfer Terasaki plates to a humidity box (any container that allows gas exchange but retains moisture) and place in an incubator.

### 3.6. Clone Expansion. Time for Completion: 14 Days

#### 3.6.1. Days 5–9 

Incubate cells undisturbed for five days but ensure that the media is not evaporating from the plates. If wells appear to have reduced volume on Day 7, carefully add an additional 5–10 μL of media to each well without disturbing the cells.

#### 3.6.2. Day 10

19. Take note of which wells have cells in them using an inverted light microscope. Clones that have cell populations that cover more than a third of the well should be transferred into a 96 well plate in 100 μL of media. Approximately 25–35% of sorted cells expand enough to be transferred.
20. Clones that have cover less than one third of the well should be left in situ until they have further expanded and add a dilution of doxycycline (dilute 1 mg/mL 1:50 and add 2 μL to the 20 μL for 2 μg/mL final concentration). Mix gently, no more than four times, with a pipette set to 15 μL and visually confirm cells have been evenly redistributed in the well. Clones that have not expanded after 15 days post-sort should be discarded (unless slow growth is a likely outcome relevant to the target locus in which case further time may be given).
  Note: If the wells contain less than 15 μL, first add the appropriate volume of complete media.

#### 3.6.3. Day 11 onwards

21. Expand the clones from 20 μL Terasaki wells → 100 μL 96 well → 200 μL 96well → 2× 200 μL 96 wells → 500 μL in 24 well → 1 mL in 24 well. Expect ~30 clones to survive this process.
22. Once the cells are confluent in 1 mL of media in the 24 well plate, prepare 1.5 mL microcentrifuge tubes labelled with a unique ID for each clone and an equivalent cryovial.
23. For each well, gently mix the cell suspension to evenly distribute the cells, then aliquot 500 μL into the cryovial and 500 μL into the matched microcentrifuge tube.
24. Add 500 μL of freezing media to the cryovial and mix by pipetting up and down once before sealing the cap and submerging the vial in a container of dry ice.
25. Add 1 mL of PBS to the microcentrifuge tube and spin at 350× *g* for 5 min.
26. Aspirate as much PBS as possible from the pelleted cells and then submerge the tube into dry ice.
27. Cryovials should be stored at −80 °C for up to four weeks and transferred to liquid or vapour phase nitrogen cryostorage thereafter.
28. DNA can be extracted from the cell pellets (as described in Section 3.2) in microcentrifuge tubes and screened by polymerase chain reaction (PCR) amplification and Sanger sequencing coupled with TIDER software [24] or restriction digest to determine correct integration of the required genomic modification. Indels may be analyzed in by combining Sanger sequencing with TIDE software [25], or amplification products may be cloned and sequenced. Next generation sequencing (NGS) is extremely useful in the analysis of HDR and indels and a novel method describing high-throughput multiplexed screening of modified clones is reported in Nussbaum et al. [26].
29. Recover the correctly targeted clones in 2× 200 μL 96 wells and check them 24 h post-thaw.
  **OPTIONAL STEP** Clones that are growing poorly can be supplemented with 2× EPO (6 IU/mL), 2× SCF (100 ng/mL) and 2× DEX (1.68 μM) (as well as 2 μg/mL DOX) every second day until they are in 2× 96 well.

## 4. Expected Results

To circumvent the lack of specific antibodies and allow detection of Codanin-1 and C15ORF41 proteins we inserted DNA sequences encoding a Strep-tag [19] and FLAG-tag [20] at the 3′ end of *CDAN1* and the 5′ end of *C15ORF41* and we show the outcome of this as expected results here. Surveyor assays for *CDAN1* sgRNAs 1 and 2 (Figure 3) show that both sgRNAs are capable of successfully inducing Cas9 activity at the correct targeting site, however, sgRNA 1 was selected because it produced a cut closest to the desired region of insertion, immediately preceding the termination codon. Similarly, for *C15ORF41* both sgRNA 2 and sgRNA 4 allowed good cutting efficiency (Figure 3), but because sgRNA 4 generated the most efficient cutting it was used for targeting.

To date we have used the protocol described to generate four lines of HUDEP-2 cells harbouring inserted sequences (Table 2 and Figure 4). These figures are given as an example and it should be noted that efficiencies are likely to vary in a locus dependent fashion. From 600 GFP positive cells sorted for each experiment, ~30 survive the initial expansion phase and of those ~20–42% contain at least one successfully targeted allele. The majority of the remaining alleles are unedited and the rest contain indels mediated by nonhomologous end joining (NHEJ). Table 2 shows the outcomes of the edited alleles. It should be noted that by performing long-range amplification of target loci we have routinely found deletions of several hundred base pairs to have been associated with clustered regularly interspaced short palindromic repeats (CRISPR/Cas9)-associated cut sites. Therefore, care should be taken when designing assays to characterise target sites. As we observe some non-genetic clonal variability we would recommend making 2 or 3 clones for each line required. Although the karyotype of the wild-type HUDEP-2 cells reported here is conserved in our modified clones there may be some variation as the chromosome counts in the clones of HUDEP-2 cells in our hands differ slightly from those reported by Vinjamur and Bauer (2018) [27].

This protocol provides detailed step-by-step instructions for achieving cutting of genomic DNA and HDR mediated integration of short donor sequences in HUDEP-2 cells using CRISPR/Cas9. HUDEP-2 cells provide an alternative model system to erythroleukaemic lines and, unlike in vitro differentiated iPSCs, express predominantly adult globins, making them a useful tool for unpicking the pathogenesis of erythroid disorders. HUDEP-2 cells double every 24–36 h allowing the generation of large quantities of material for downstream analyses previously impeded by low cell numbers. HUDEP-2 cells also provide an isogenic background, which circumvents variable patient phenotypic abnormalities. This protocol can be applied to additional immortalized erythroid cell lines as they become available (e.g., Ref. [28]) and provides a means to efficiently generate modified cell lines.

Our approach takes advantage of recent studies that identify short ssODNs as a useful source of template for HDR [29,30]. If larger fragments are required for HDR then use of a delivery vector may facilitate this, see Reference [13]. HUDEP-2 cells also have scope for the use of longer linear double stranded donors, which have been efficiently integrated in HEK293T and human embryonic stem cells, particularly when paired with cell synchronization [31]. Treatment with Nocodazole and Cyclin-D1 causes cells to accumulate in the S/G2/M phase where HDR is the favoured pathway for DNA repair [31,32], although it remains to be shown whether this will work as efficiently in HUDEP-2 cells. Donors with an antibiotic resistance gene or that encode a fluorescent protein provide a means of selection at the level of integration and would also increase the rate of modification. Recently, modified *S. pyogenes* Cas9 proteins have been generated that show reduced off-target cutting events while preserving a high level of activity at on-target sites (e.g. [33,34]). These proteins offer a useful alternative where the user wishes to use RNP editing of erythroid cells as described in Reference [13].

A major consideration when utilizing the CRISPR/Cas9 system is that the enzyme tolerates mismatches in both the PAM and the protospacer element [35,36,37,38,39,40]. Although off-target activity of Cas9 is more concerning in a clinical context than when generating model systems, non-specific cleavage could lead to off-target modifications. In this study we used a short guide of 18 nucleotides to minimize the homology available for off-target binding [41]. It should be noted that shorter protospacer sequences can lead to a drop in editing efficiency, if low efficiency is an issue then longer protospacers of 20 or 21 nucleotides should be considered. Off-target sites can differ from the targeted locus by up to seven nucleotides [39,42] and these can be in the form of single nucleotide mismatches, small indels or DNA-RNA bulges [33]. However, off-target activity generally decreases with increasing numbers of mismatches and three mismatches have been reported to ablate almost all off-target activity [35,38,42]. Off-target activity varies according to cell type and transfection conditions [38,43,44,45] and there are no simple rules that can be applied to its prediction [38,42]. However, unbiased detection of off-target effects may be determined by Digenome-seq [40] or CIRCLE-seq [45] alternatively predicted off-target sites may be amplified using specific primers followed by mismatch detection or sequencing. Where necessary, perhaps the most effective way to navigate this limitation of the Cas9 system is to generate the same mutation using two different guides and compare the resultant cell lines. This would help to distinguish phenotypic abnormalities arising from the intended modification from the possible effects of off-target cleavage. This would increase the likelihood that phenotypic abnormalities arise due to modification alone and not the effect of off-target cleavage and clonal variability.

**Figure 5:**
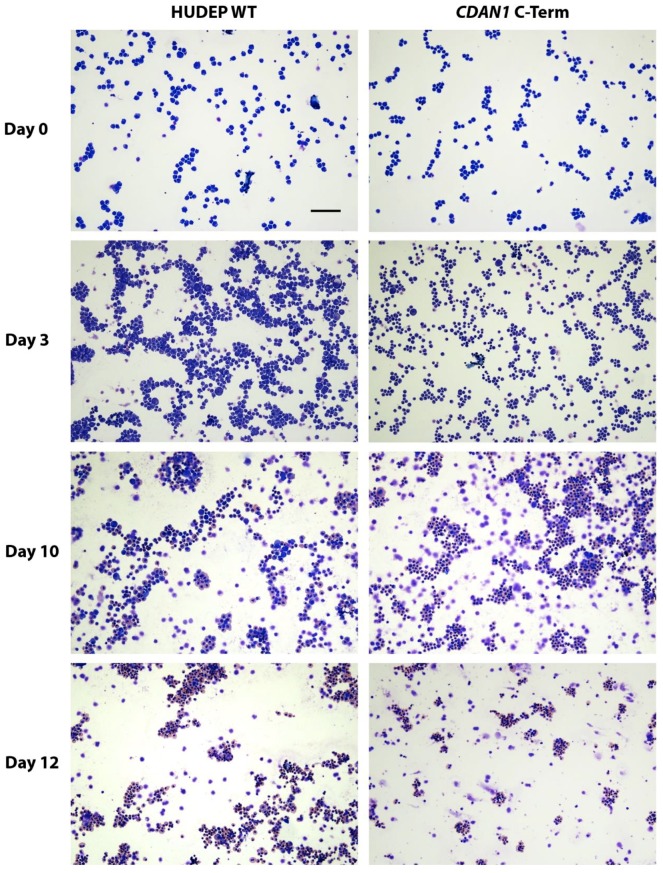
Differentiating HUDEP-2 erythroblasts stained using a modified Wright’s method. Differentiation of unmodified HUDEP-2 cells (HUDEP Wild Type WT, **left column**) and HUDEP-2 cells containing *CDAN1* tagged at the C-terminus with Strep-II (**right column**) is equivalent at each stage shown. The scale bar is 100 μm.

Immortalized cell lines are a useful resource for unpicking the relationship between patient genotype and disease phenotype as long as their assays are interpreted within the limitations of an immortalized system. As such, we karyotyped the HUDEP-2 cells using the Infinium Omni5-4 v1.2 CGH array and found that they have a modal chromosome count of 51. These cells have relatively fewer abnormalities than HEL or K562 cells [4], which also have whole-chromosome duplication events with modal chromosome numbers of 66 [2] and 67 [46] respectively. In vitro differentiation of the cells confirmed that despite the potential off-target effects of the genome editing process, each clone still followed a normal differentiation process and produced mature erythroblasts (Figure 5).

Since their inception in 2013, HUDEP-2 cells have been widely utilised in conjunction with the CRISPR/Cas9 system. However, few details are available for the modification of these cells, especially where methods alternative to lentiviral transduction are preferable [9,47,48,49,50,51]. Here we describe a detailed protocol, similar to the strategy employed in Reference [9], for the efficient modification of immortalised erythroid progenitor cells in an easy to follow and scalable method. In our experience, HUDEP-2 cell differentiation can provide adult-globin expressing erythroblasts in numbers 4–7 fold higher than starting cell numbers within 9–12 days. These cells provide an alternative to existing immortalised erythroleukemia lines and support the need to replace animal models [6].

## Figures and Tables

**Table 1:** Copy number variation in HUDEP-2 Cells.

Chromosome	Start (hg18 *)	End (hg18)	Event	Length	Cytoband	Count of Genes
3	46,990,391	50,479,675	ROH	3,489,285	p21.31	121
3	156,276,396	159,447,085	ROH	3,170,690	q25.31–q25.33	24
6	0	171,115,067	CN Gain	171,115,068	p25.3–q27	1,433
8	0	83,821,458	CN Gain	83,821,459	p23.3–q21.13	597
8	83,821,459	110,503,487	CN Gain	26,682,029	q21.13–q23.2	165
8	110,503,488	125,424,290	CN Gain	14,920,803	q23.2–q24.13	65
8	125,424,291	138,306,862	CN Gain	12,882,572	q24.13–q24.23	76
8	138,306,863	146,364,022	CN Gain	8,057,160	q24.23–q24.3	141
14	37,393,981	37,510,754	CN Loss	116,774	q13.3	2
17	30,301,223	81,195,210	CN Gain	50,893,988	q11.2–q25.3	1,022
18	0	15,375,878	CN Loss	15,375,879	p11.32–p11.21	119
18	18,561,020	78,077,248	CN Gain	59,516,229	q11.1–q23	310
19	0	59,128,983	CN Gain	59,128,984	p13.3–q13.43	1,872
21	14,368,320	48,129,895	CN Gain	33,761,576	q11.2–q22.3	363

* Genome build NCBI36/hg18 was used. ROH: Region of homozygosity; CN: copy number.

**Table 2:** Targeting efficiency of edited alleles.

Locus	% Alleles Repaired by NHEJ	% Alleles Targeted by HDR
*CDAN1* C-Term	80%	20%
*C15ORF41* N-Term	65%	35%
*C15ORF41* C-Term	70%	30%
*ATRX*	58%	42%

NHEJ: Nonhomologous end joining; HDR: homology directed repair.

## Appendix A. aCGH Analysis

Array comparative genomic hybridization (aCGH) data were processed using GenomeStudioV2009.2 (Illumina, Inc., San Diego, CA, USA) and analysed using Nexus 8.0 Discovery Edition (BioDiscovery, Inc., El Segundo, CA, USA) and the following settings single nucleotide polymorphism (SNP) Rank Segmentation (based on circular binary segmentation statistics): Significance Threshold = 1.0 × 10^−6^, Max Contiguous Probe Spacing (Kbp) = 500.0, Minimum number of probes per segment = 10, High Gain = 0.5, Gain = 0.12, Loss = −0.12, Big Loss = −0.7, 3:1 Sex chromosome gain = 1.2, Homozygous Frequency Threshold = 0.98, Homozygous Value Threshold = 0.85, Heterozygous Imbalance Threshold = 0.46, Minimum loss of heterozygosity (LOH) Length (Kb = 500, Minimum SNP Probe Density (Probes/Mb = 0.0. Flagged calls were inspected visually and curated to exclude common variants noted in the Database of Genomic Variants [52], regions of homozygosity (ROH) <2 Mb and events documented previously in unrelated datasets in house.
